# Outbreaks of Virulent Infectious Bursal Disease in Flocks of Battery Cage Brooding System of Commercial Chickens

**DOI:** 10.1155/2016/8182160

**Published:** 2016-08-11

**Authors:** H. B. Aliyu, L. Sa'idu, A. Jamilu, A. D. Andamin, S. O. Akpavie

**Affiliations:** ^1^Veterinary Teaching Hospital, Ahmadu Bello University, P.M.B 1045, Zaria, Nigeria; ^2^Deen Farm, Km 3.7, Kaduna International Airport Road, Kaduna, Nigeria; ^3^Department of Agricultural Technology, Federal College of Horticultural Technology, P.M.B. 108, Dadin Kowa, Gombe, Nigeria; ^4^Department of Veterinary Pathology, Faculty of Veterinary Medicine, Ahmadu Bello University, P.M.B 1045, Zaria, Nigeria

## Abstract

Clinical and pathological investigations were conducted on outbreaks of infectious bursal disease (IBD) in pullets under brooding using the battery cage system in a commercial poultry farm in Kaduna, Nigeria. Two consecutive outbreaks of IBD on the same farm were studied. The onset of the disease and morbidity and mortality rates were recorded. Postmortem examinations were conducted and gross lesions recorded. Tissues were collected and fixed in 10% buffered formalin and processed for histopathological examinations. In the first outbreak, 80 to 100% of the chicks were affected at the age of 4 to 5 weeks and mortality rate was 95.8% and lasted for 9 days. In the second outbreak, the mortality rate was 43.3% and it also lasted for 9 days. At the onset of the disease, the birds were also 4-week-old like in case 1. The disease was diagnosed based on clinical signs, pathology, and agar gel immunodiffusion test (AGID). Clinical signs, gross lesions, and histopathological findings were characteristic of virulent infectious bursal disease. After the first outbreak (case 1) the house was disinfected using polidine® (iodophor compound), V-ox® (inorganic peroxygen compounds), CID_20_® (quaternary ammonium chloride, aldehydes, and alcohol), terminator III® (phenols), and glutasan® (aldehyde and quaternary ammonium chloride). But they failed to eliminate the IBD virus from the poultry pen.

## 1. Introduction

Infectious bursal disease (IBD) is an acute, highly contagious viral disease of young chickens that primarily affects lymphoid tissues [1–3]. It is caused by a member of the genus* Avibirnavirus* in the family Birnaviridae [4, 5]. The disease was first described by Cosgrove in [6] around Gumboro, Delaware, USA.

Ojo et al. [7] first described the disease in South Western Nigeria and was confirmed by Onunkwo in [8]. Since then several studies have shown that the disease is of major concern to the poultry industry in the country [9–12]. Infectious bursal disease virus (IBDV) has tropism to actively dividing precursor B lymphocytes, primarily in the bursa of Fabricius, but other immune organs are also involved [13].

The disease is mainly controlled by rigorous sanitary measures and vaccination through the use of either live or killed vaccines. Infectious bursal disease intermedite or intermediate plus vaccines are commonly used to protect broilers and commercial pullets replacements from field IBDV exposure [14–18]. Despite vaccinations outbreaks of IBD are occasionally reported in vaccinated flocks of broiler and pullets with varying degree of mortality rates. Reversion of these attenuated vaccinal strains to more virulent phenotypes under field and experimental conditions has been frequently reported [19, 20] possibly due to a lack of IBDV polymerase fidelity during vaccine viral genome replication in the host cells.

Infectious bursal disease virus is very stable and has been reported to resist many disinfectants at certain concentrations and or conditions [3, 21]. The virus remained viable by exposure for 1 hour at 30°C to 0.5% phenol and 0.125% thimerosal [3]. Landgraf et al. [22] found that the virus survived 60°C but not 70°C for 30 minutes, and exposure to 0.5% chloramine after 10 minutes and 0.5% formalin for 6 hours destroyed the virus. In addition, iodine complex had deleterious effects on the virus at 23°C for 2 minutes [3].

Certainly, the hardy nature of this virus is one reason for its persistent survival in poultry houses even when thorough cleaning and disinfection procedures are followed [3, 23]. There are several claims by some practicing veterinarians and animal scientists that disinfectants like hypo, V-ox, and iodine are effective for the control of virulent IBD. Currently, there is no report available on the efficacy of these agents as treatment regimen for IBD in field or clinical trials in Nigeria. This paper describes the clinicopathological correlation of virulent IBD outbreaks in two successive flocks of commercial chickens raised under battery cage system in attempted treatments.

## 2. Materials and Methods

### 2.1. Study Area and Case History

Outbreaks of suspected IBD case were reported to the Avian Clinic of Veterinary Teaching Hospital, Ahmadu Bello University, Zaria, from a commercial poultry farm in Kaduna metropolis located at Km 3.7, Kaduna International Airport Road, Kaduna. Two subsequent outbreaks of acute IBD in pullets raised under battery cage system in the same farm were investigated.

### 2.2. Clinical Assessment and Postmortem Examination

Repeated farm visits were made and analysis was made on the clinical presentations of the disease, farm records including source of the chicks, breed, vaccination, age, major signs observed, intervention, and the mortality rate. Moribund and dead birds were collected and thorough postmortem examination was conducted. The gross lesions were recorded.

The first outbreak was encountered in a flock of 8,413, 4.3-week-old brown pullets (ISA brown). The flock had received Newcastle disease vaccines at days 10 and 20 of age and IBD vaccines at days 8, 18, and 31 of age using Bioveta® Ornibur (intermediate strain, Bioveta, s.r.o. Ltd., Czech Republic) and B2K Indovax® (invasive intermediate strain, B.P. Vet., India) vaccines at 10 mL reconstitution per bird per os, respectively. Clinical signs such as dullness and ruffled feathers were observed at about 5 hours after Bursa B2K Indovax IBD vaccine administration. Twenty birds were lost on the first day and immediately Floricol® (Florfenicol, VIC Animal Health, Russia) at 1 mL/L of water was given for a day and V-ox (mixture of inorganic peroxygen compounds; Polchem Hygiene Laboratories Pvt. Limited, India) at 1 g/L of water, Vitavet® (multivitamins, vitamins A, Bs, E, D, and K, Pharma-Swede, Egypt) at 1 g/2 L of water, and glucose at 1 g/L of water were administered for 3 days. On days 2 and 3 of the outbreak, Floricol was changed to doxy-gen® (doxycycline and gentamycin, Kepro, Holland) at 1 g/2 L of water and on day 4 doxy-gen was switched to oxyfuravit® (Oxytetracycline HCl, Furaltadone HCl, vitamins (A, B2, B12, C, D3, E, and K3), Nicotinic Acid, DL-Methionine, and Lysine, Maridav, Ghana) at 1 g/L of water and substituted V-ox for polidine (iodine, alkylphenoxypolyglycol ether and phosphoric acid; Animal Care Services Konsult, Nigeria) at 1 mL per 2 liters of water for 5 days. Despite medications, the morbidity and mortality worsened and lasted for 9 days. The birds that survived (347) were disposed off and the house was thoroughly cleaned, washed, and disinfected.

The second outbreak occurred in a flock of 9,000, 4-week-old brown pullets. The chicks were vaccinated against ND at days 10 and 20 of age and IBD at days 8 and 18 of age using ABIC® IBD vaccines through oral and intranasal routes, respectively. During the outbreak, oxyfuravit was administered in feed and water at the rate of 10 g/5 kg and 1 g/L, respectively, together with polidine at 1 mL/L. V-ox, Neoceryl plus® (neomycin, erythromycin, oxytetracycline, colistin sulphate, streptomycin, and multivitamins, Alfasan International BV, Holland) at 1 g/L, and Tylodox (tylosin tartrate and doxycycline; Kepro, Holland) at 1 g/L together with multivitamins were administered for 3 days each.

However, despite the interventions, the morbidity and mortality also lasted for 9 days. Postmortem examination and sample collection were carried out in both cases.

### 2.3. House Cleaning and Disinfection

After the first outbreak, the house was rigorously cleaned, washed, and disinfected using CID_20_ (alkyldimethoylammonium chloride, glutaraldehyde, formaldehyde, glyoxal, and isopropanol; CID Lines, leper-Belgium), at dilution of 1 : 200, glutasan (glutaraldehyde and alkyldimethoylammonium chloride; Pine Oil, USA), at 1 : 400 of water, terminator III (ortho-phenylphenol, ortho-benzyl-para-chlorophenol, and para-tertiary-amylphenol; Neospark, India) at 2 mL/L of water, and hypo® (sodium hypochlorite; Multipro Enterprises Ltd., Nigeria) at 2 mL/L of water alternately. The house was left fallow for 40 days before restocking.

### 2.4. Sample Collection and Processing

Tissues were collected for virological, bacteriological, and histopathological examination. Bursae of Fabricius (BFs) were aseptically harvested into universal bottles and stored at −20°C for viral detection. Liver was sent for bacterial culture and identification. Tissues including skeletal muscles, spleen, BF, and kidneys were fixed in 10% neutral buffered formalin for histological examination. The fixed tissues were processed routinely for histopathology stained with haematoxylin and eosin and examined with the light microscope.

Frozen samples of the BFs were homogenised into 50% w/v suspension in phosphate buffered saline (PBS). The homogenate was centrifuged at 2000 rpm for 30 minutes and the supernatant was harvested and tested for IBD virus using AGID test as described by OIE [14].

## 3. Results

### 3.1. Clinical Evaluation

**Figure 1:**
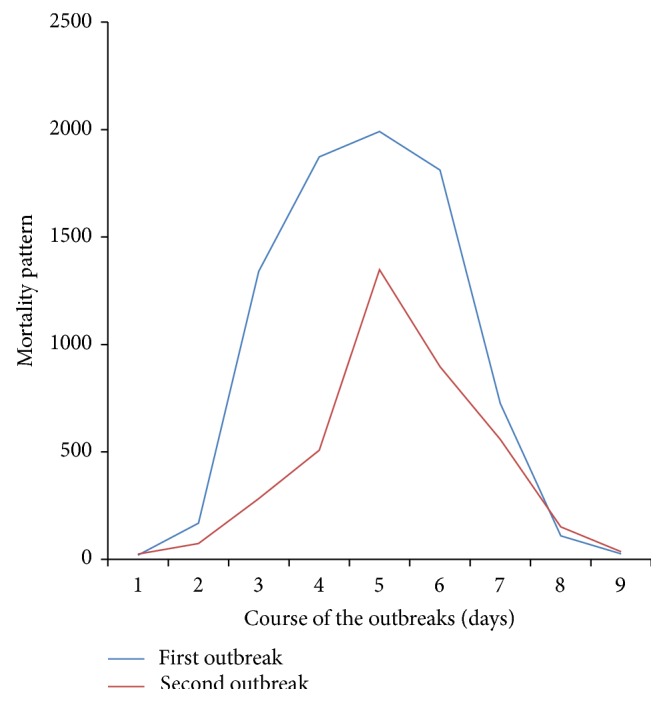
Mortality pattern in the two outbreaks of virulent infectious bursal disease in pullets on battery cage system.

Clinical signs observed in chicks in the two outbreaks were ruffled feathers, depression, hurdling together, anorexia, prostration, and whitish diarrhoea. Mortality recorded spiked within 5 days of onset and then declined but lasted for 9 days in both cases. Morbidity and mortality recorded were 100% and 95.8%, respectively, for case 1 and 80% and 43.3%, respectively, for case 2. The mortality patterns are shown in Figure 1.

### 3.2. Postmortem Findings

**Figure 2:**
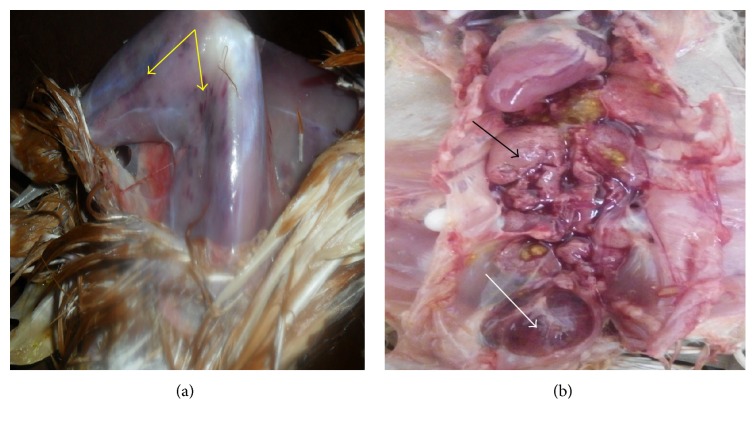
(a) Ecchymotic haemorrhages on the thigh and leg muscles (yellow arrows), (b) enlarged pale kidneys (black arrow), and edematous and haemorrhagic bursa of Fabricius (white arrow) of 4-week-old brown pullets from case 2.

The carcasses were in good condition but moderately dehydrated. There were petechial and ecchymotic haemorrhages on the pectoral, thigh and leg muscles and caecal tonsils and at the junction between proventriculus and ventriculus. The liver was severely congested and the spleen was enlarged and mottled but in some cases atrophied. In most cases the bursa of Fabricius was edematous and haemorrhagic with yellowish gelatinous exudate on the mucosal surface. The kidneys were swollen and pale (Figure 2).

### 3.3. Histopathological Findings

**Figure 3:**
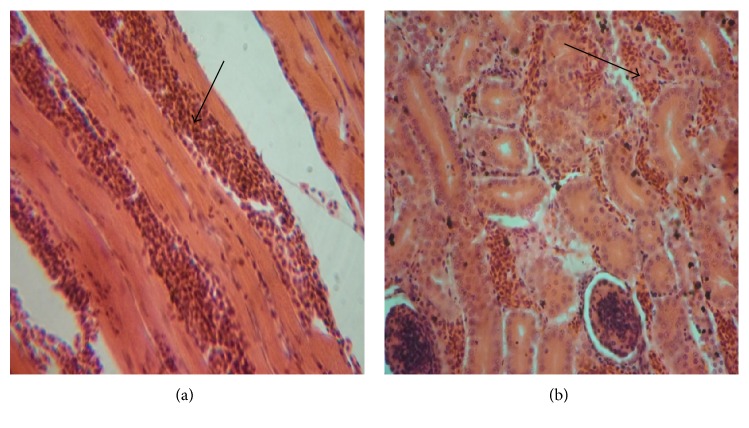
Photomicrographs of haemorrhage (arrows) in muscle (a) and kidney (b) of 4-week-old chicks affected with infectious bursal disease. H & E ×200.

**Figure 4:**
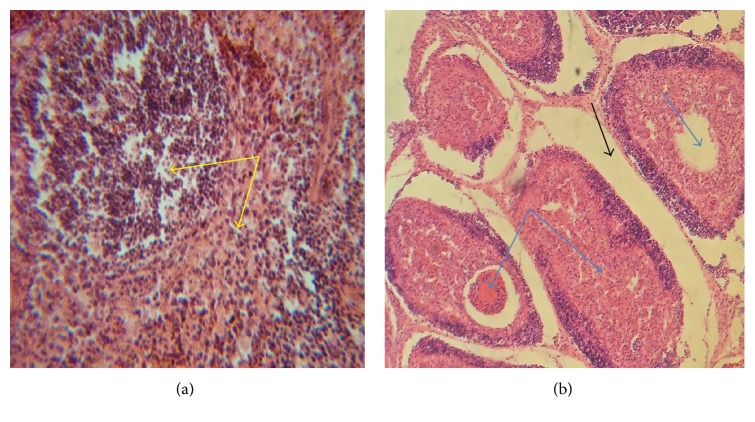
Photomicrographs of 4.3-week-old chicks affected with infectious bursal disease showing moderate lymphocytes depletion in the lymphoid nodules (yellow arrows) of the spleen (a), marked interfollicular oedema (black arrow), and cystic cavitation and necrosis (blue arrows) in the medullar of bursal follicles (b). H & E ×200.

Microscopic examination of tissues showed moderate haemorrhages in the muscles and kidneys (Figures 3(a) and 3(b)) and the spleen showed moderate lymphoid depletion in the lymphoid nodules (Figure 4(a)). There was marked interfollicular oedema and depletion of lymphocytes from the lymphoid nodules in the BFs (Figure 4(b)). Other lymphoid nodules of the BF showed degeneration and necrosis of lymphocytes and cystic cavitations with heterophil infiltrates (Figure 4(b)).

### 3.4. Bacteriological and Virological Examinations

*Escherichia coli* were isolated from the liver and the bursal homogenate gave positive reactions to the IBDV known antiserum.

## 4. Discussion

The purpose of this investigation was to determine whether polidine or V-ox at 1 mL/L could significantly reduce typical IBD lesions in chicks infected with IBDV. However, the clinical manifestations and gross lesions observed in this study are similar to those reported previously [12, 24–32] that chickens infected with IBDV exhibit anorexia, prostration, and white diarrhoea while grossly the BFs appear yellowish, hemorrhagic, and turgid with prominent striations, oedema, and caseous material found and varying degrees of hemorrhages in the thigh and breast muscles and at the junction between gizzard and proventriculus. The microscopic lesions seen in this study are similar to those reported [3, 12, 25, 27, 28, 32–38] that found that bursae from IBDV exposed birds showed lymphoid depletion in the bursal follicles, interfollicular oedema, cellular debris in the medullary areas with necrosis, and/or eosinophilic cystic cavitations. These typical lesions confirmed that the attempted treatment claims cannot stand for control of IBD.

The observed morbidity and mortality are suggestive of vIBD and agree with the report by Asif et al. [39], Mbuko et al. [12, 28], El-Mahdy et al. [27], and Ezeibe et al. [40] that chickens infected with virulent IBDV could experience high morbidity rate of 80–100% and mortality rate of 40–90% depending on the presence of secondary bacterial complication. The sudden onset, high morbidity, spiking mortality pattern, and sharp recovery from clinical signs are suggestive of the disease. However, the course of the disease lasted longer than what was reported by Cosgrove [6], Cho and Edgar [41], Okoye and Uzoukwu [10], Mbuko et al. [12, 28], and Ezeibe et al. [40] that IBD runs its full course in about 7 days. During these outbreaks, mortality lasted for 9 days and peaked at day 5 in both cases. This could be attributed to the management system (battery cages) of the birds. The pullets in these cases were brooded under battery cages which provide minimum contacts of the chicks with one another and their droppings. Although aerosol route of the disease transmission exists, faeco-oral route is the major route by which susceptible chick can be infected [30]. The disease might spread very fast in deep litter management system due to free contact of the infected and noninfected birds. Also in the deep litter, the birds have direct access to their droppings, and as such the feed and water can be contaminated by the droppings of infected birds as suggested by Saif [42] and Eterradossi and Saif [3].

The high mortality recorded in the first outbreak may be due to the fact that it was the first outbreak of the disease in the farm and also the type of vaccines and vaccination programme employed may not have protected the birds against the field IBD virus. There is the likelihood that the intermediate vaccines administered at days 8 and 18 were interfered by maternally derived antibodies (MDAs) and therefore the chicks were unprotected and the intermediate plus vaccine given at day 31 may have exacerbated the condition. Several studies have indicated that high MDAs at the time of IBDV vaccination might interfere with the vaccine response, neutralise the vaccine virus, and delay or even prevent the induction of humoral immunity [15, 33, 43–45]. However, virulent strains of IBDV of same serotype have been reported to overcome high MDAs in commercial flocks vaccinated with vaccines developed from different variants, causing up to 60% to 70% mortality [46]. Although vaccination of chickens has remained the principal method to control this disease [27, 47], the effectiveness of vaccinations relys on variants of the virus circulating in the area [40]. Adamu et al. [48] studied the relationship between field and foreign vaccine strains in Nigeria and reported that when IBDV strains spread from their region of origin to a different region they mutate alongside indigenous field strains. The antigenic differences between field and vaccine viral strain could be responsible for vaccine failures. Therefore, vaccines being used in the country should be those made from strains of the virus circulating in Nigeria.

The source of the second outbreak for the subsequent batch of pullets may be due to the persistence of the virus in the environment between outbreaks, since IBD virus is very stable and resistant to many disinfectants [21, 42]. It is also pertinent to note that the brooding house was thoroughly cleaned and disinfected using the following disinfectants (polidine, V-ox, CID_20_, terminator, and glutasan) following the first outbreak before restocking. This further confirms previous report that once an outbreak of IBD occurs in a farm it will continue to occur with reduced mortality [3, 49] as seen in this investigation.

The* Escherichia coli* isolated from the bacteriological investigation was not surprising due to the immunosuppressive effects of the IBDV [50]. Consequences of immunosuppression due to IBD include poor response to vaccinations, gangrenous dermatitis, inclusion body hepatitis-anaemia syndrome, and* Escherichia coli* infection [51, 52].* Escherichia coli* is known to be the abundant normal flora of gastrointestinal tracts of poultry. Part of the mechanism of immunosuppression in IBD is lymphocyte lysis and apoptosis [3].

There are several claims by field veterinarians and animal health workers that certain disinfectants (e.g., iodine, sodium hypoclorite, and V-ox) when given orally are effective for the treatment of IBD. In these cases, the chemicals administered seemed not to have been effective as the disease ran its normal course with a very high mortality rate in case 1 and mortality rate of about 40% in case 2.

## 5. Conclusion

In conclusion, the IBD vaccines currently being used to vaccinate birds against IBD in Nigeria may be antigenically different from the IBD virus circulating in our environment. There is therefore the need for effective sanitary measures and adequate decontamination with sufficient fallow period before restocking of birds after an IBD outbreak in a poultry farm. The findings of this study have shown that polidine and V-ox at 1 mL per litre of water given orally in chickens will not prevent typical IBD lesions in case of field exposure. Also, effort should be made to produce vaccine locally with the strains of the circulating IBD virus.
